# Reshaping the Healthcare Sector with Economic Policy Measures Based on COVID-19 Epidemic Severity: A Global Study

**DOI:** 10.3390/healthcare10020315

**Published:** 2022-02-07

**Authors:** Timotej Jagrič, Dušan Fister, Vita Jagrič

**Affiliations:** Institute of Finance and Artificial Intelligence, Faculty of Economics and Business, University of Maribor, Razlagova 14, SI-2000 Maribor, Slovenia; timotej.jagric@um.si (T.J.); dusan@dusanfister.com (D.F.)

**Keywords:** COVID-19 pandemic, healthcare sector transformation, research and development, artificial intelligence, economic development, health system resilience

## Abstract

Governments around the world are looking for ways to manage economic consequences of COVID-19 and promote economic development. The aim of this study is to identify the areas where the application of economic policy measures would enhance the resilience of societies on epidemic risks. We use data on the COVID-19 pandemic outcome in a large number of countries. With the estimation of multiple econometric models, we identify areas being a reasonable choice for economic policy intervention. It was found that viable remediation actions worth taking can be identified either for long-, mid-, or short-term horizons, impacting the equality, healthcare sector, and national economy characteristics. We suggest encouraging research and development based on innovative technologies linked to industries in healthcare, pharmaceutical, and biotech, promoting transformation of healthcare systems based on new technologies, providing access to quality healthcare, promoting public healthcare providers, and investing in the development of regional healthcare infrastructure, as a tool of equal regional development based on economic assessment. Further, a central element of this study, i.e. the innovative identification matrix, could be populated as a unique policy framework, either for latest pandemic or any similar outbreaks in future.

## 1. Introduction

The COVID-19 pandemic is a case of a health-triggered economic crisis resulting in a simultaneous health and economic crisis [1]. Besides the healthcare sector operating on its limits, there is not a single economic sector left not being impacted by COVID. The measures to slow-down or control the spread of virus impacted the daily life of households and, what is more, caused economic costs. The closures of public life and constrains on people’s mobility caused many businesses to lose revenue in the sort-run and are fearing the loss of customers due to changed consumers’ habits in the middle- and long run. There were disrupted supply chains causing delay in production and delivery of goods [2]. Besides the initial economic shock, together with simultaneous demand and supply disruptions, the COVID-19 pandemic was of a size not experienced before, and economic consequences could even lead to long-lasting declines in global economic output [3].

Not all applied measures turned out to be effective. As reported by Berry [4], for the first wave of the pandemic the effects of shelter-in-place (SIP) orders did not exhibit a detectable impact on disease spread or COVID-19 caused deaths.

As seen by Sagan et al., 2021 [5], for the case of four European countries, and similarly in many countries, there were expensive but effective measures in containing the spread of the virus, such as the lockdown measures. The applied measures have hidden the insufficiency and the unpreparedness of the healthcare systems to manage the health crisis.

Traditional measures to tackle epidemics, although efficient (e.g., quarantine, social distancing, mobility restrictions, economic lockdown, etc.), have, in modern societies, two main problems: the discussion on restricting human rights, and immense financial burden.

Regarding the discussion on human rights, there are already several findings. Protecting public health requires prioritizing the common good and broader societal implications over individual autonomy and the interest of an individual. Further, protecting an individual’s health also includes preventing him from contacting diseases, resulting in long-term interests prevailing over short-term interests. Therefore, public health policies are designed in line with the view that human health is given priority over human rights, as stated by Chia and Oyeniran in 2020 [6]. Besides the negative impact of applied measures against the spread of the virus on human rights, these was also an undesirable effect on the security of food and water, as reported for the African region by Boretti [7]. Further, common measures in the epidemic were not sustainable [7]. The evidence reported by Huffstetler et al. [8] on public health actions across six geographic regions shows an impact on distinct human rights and on civil, political, economic, and social rights that underlie public health. Additionally, Huffstetler et al. [8] found disproportion in effect on the human rights of particular groups, such as women and minority populations.

Undoubtedly, COVID-19 patients filled the healthcare capacities and caused access and quality of healthcare to worsen for many other patients, which can be seen in greater numbers of avoidable deaths caused by diagnostic delays. Apart from great human loss, diagnostic delays also bring economic consequences, as shown in the case of England by Gheorghe et al. [9]. Authors estimate productivity losses of GBP 104 million over 5 years, and this figure only reflects the first epidemic wave caused by additional excess cancer deaths due to diagnostic delays [9].

The economic cost of the pandemic, consequently, was urging for reasonable measures to promote economic recovery. Guerrieri et al. [10] argue that economic shocks associated with the COVID-19 epidemic may be a kind of a supply shock that triggers changes in aggregate demand larger than the shocks themselves. Due to healthcare sector capacity limits, urgent measures were applied, many of them harming the economy. In cases of less prepared health systems, governments had to apply stricter confinement measures and higher levels of stringency in the confinement measures, which have larger negative, socio-economic effects [11]. Therefore, more resilient healthcare systems should be developed in the future to be better prepared to handle public health crises.

Empirical results indicate that short-term economic losses were greater where less fiscal stimulus was implemented, and where monetary policy easing was limited [10]. We argue that economic policy measures for successful recovery should take into account characteristics of economic sectors. In the economic ecosystem, the healthcare sector plays an important role. The economic impact and economic characteristics of the healthcare sector were broadly explored in the literature [12,13,14,15,16,17].

Further, the literature on the economic consequences of the COVID-19 pandemic is broad and thorough [3,4,9,11,18,19,20,21,22,23,24,25,26]. Economic downturn of one country, e.g.,the U.S., will have different spill-over effects on other economic ecosystems, e.g.,the European Union, as suggested by Wang and Han [18]; no economy is isolated due to global interconnections and thus cannot avoid the economic impact of the pandemic from abroad (Chudik et al. [3]). Additionally, destabilised and disrupted supply chains due to the COVID-19 pandemic might have secondary ripple effects on other economies [23]. The literature reports on the relationship between pollution and COVID-19 related deaths while economic growth has contributed to build-up of pollutants [21].

Empirical findings have shown that healthcare sectors have large and positive macroeconomic impacts on domestic economy (e.g., Stuckler et al. [27]). The economic impact is often above the average of national economy, suggesting that this sector is a more favourable choice for economic policy. Findings also indicate (Jagrič et al. [16]) that additional spending for healthcare services stimulates the creation of jobs across the national economy and that creating jobs in the economy is higher in less developed economies. Investments into healthcare in regions (or countries) with lower regional GDP per capita will promote equality, stimulate regional output, diminish regional unemployment, and increase income levels. Investments into less developed regions suggest higher multiplicative effects, suggesting the use of investment into regional healthcare sectors as a tool for equal regional development.

Although healthcare spending has been growing for decades in a large number of countries, there were attempts to cut the costs, especially in times of public finance constraints. Empirical data and the literature give grounds to believe this strategy does not bring the desired result in either the economic perspective, as austerity measures do not promote but rather harm the recovery (Darvas et al. [14]), nor in the health outcomes, as the avoidable mortality can be affected. A study by Arcà et al. [28] reveals that, even in countries with relatively low avoidable mortality, spending cuts in healthcare can hurt survival. Furthermore, the procyclicality matters, as reducing procyclicality of government health expenditure by keeping them in bad times may generate substantial health gains (Liang and Tussing [29]).

The literature extensively explores economic effects of the pandemic along with the policy measures to reduce them and the damage to the national and global economy. These measures arise from monetary, macroprudential, and fiscal policies. Applied policies include relief measures, recovery policies, and international coordination measures and are stated to reduce the consequences independently or as a combined mix of measures [19]. However, while such a research approach explores policy options to act against the consequences of an economic crisis caused by the pandemic, our approach is innovative in moving the perspective to the options of economic policy to reduce contributing factors of the severity of the pandemic outcome. The present study is thus original in the following ways. While the economic literature often takes the perspective of empirically exploring an individual determinant or some determinants which are ex-ante, selected based on theoretical grounds and the impact on the health outcomes during a specified time frame, we take an innovative point of view. We await to identify areas where an impact on the health outcomes in the case of the COVID-19 pandemic originated and can be affected by economic policy measures in the short- or long-term perspective to enhance reliance to possible future health crisis.

The paper is organized as follows. After highlighting the relevant economic characteristics and exploring grounds for economic recovery in the first section, we present the data sources and methods used in the study. Next, Section 3 gives technical results and their interpretation regarding the research question. Finally, Section 4 and Section 5 complete with the discussion and conclusions, respectively.

## 2. Materials and Methods

Although the COVID-19 epidemic is not yet over, already a lot of data is made available by statistical offices, international organizations, national governments and their public health institutes, and many other organizations. Initially, we have collected 171 data variables for 197 countries, from 2017 to 2020, to ensure that, in some minor cases where the most current data were not available, the latest possible data, or an estimation, were taken. Collected data considered economic, infrastructure, cultural, health, and other areas. Economic variables were obtained from World Bank Open Data (https://data.worldbank.org/, accessed on 10 December 2020), IMF’s World Economic Outlook Database (https://www.imf.org/en/Publications/WEO/weo-database/2020/October, accessed on 10 December 2020), Trading Economics portal (https://tradingeconomics.com/indicators, accessed on 10 December 2020), and FDI Attractiveness Index website (Ben [30], accessed on 10 December 2020) (http://www.fdiattractiveness.com/ranking-2020/, accessed on 10 December 2020). Infrastructure variables were fetched from Enerdata (https://yearbook.enerdata.net/, accessed on 10 December 2020) and ITU (https://www.itu.int/en/ITU-D/Statistics/Pages/stat/default.aspx, accessed on 10 December 2020), while other relevant cultural variables from Wikipedia, ETH’s KOF (https://kof.ethz.ch/en/forecasts-and-indicators/indicators/kof-globalisation-index.html, accessed on 10 December 2020) (Gygli et al. [31] and Dreher [32]), and Google Mobility (GM) website (https://www.google.com/covid19/mobility/, accessed on 10 December 2020). As GM data were reported as high frequency (daily) data, basic transformation for integration with the low-frequency data (others) were necessary. First, the average values of GM data during the first corona-virus outbreak (1 March–1 May 2020) and during the second outbreak (last two months prior to 6th December 2020) were calculated. Two vectors of six categories (retail and recreation, supermarket and pharmacy, parks, public transport, workplaces, and residential) were built in this way.

Next, the average between the two built vectors was taken to form a single, consolidated, composite indicator. There, it was found that the retail and recreation category showed as most relevant here. Variables on health outcomes were obtained from WHO’s Global Health Observatory data repository https://apps.who.int/gho/data/node.main (accessed on 10 December 2020) and Nextstrain https://nextstrain.org/ncov/global (Hadfield et al. [33], accessed on 10 December 2020). These were also categorized as high frequency data (number of infected, dead, and recovered people and number of clade mutations) and were recorded at the day of beginning the research, i.e., 10th December. Again, these required specialized treatment, such that composite indicators were built. The rest of the variables came from a consolidated web portal, Our World in Data https://ourworldindata.org/charts (accessed on 10 December 2020), which holds datasets of different data providers. After building a complete (consolidated) dataset as a combination of high and low frequency data, missing data were found such that cleaning of dataset was necessary. Two versions of reduced datasets were generated. In the first, there were data for 78 countries with 11 variables altogether. In the second, by reducing the number of countries, 13 more variables could be included. The list of the explanatory variables is as follows and can be divided into several groups: virus characteristics (COVID-19 cases—cumulative total, COVID-19 virus clade 20A, and COVID-19 virus clade 20B), population characteristics (share of population older than 65, share of the population living in urban areas, mean BMI (male and female)), equality characteristics (female employment-to-population ratio and Gini index of consumption), healthcare sector characteristics (share of public healthcare sector and the Healthcare Access and Quality Index), national economy characteristics (GDP per capita and PPP, i.e., constant 2011 international $, High-Tech export (share of manufactured exports), FDI country attractiveness, and share of the agriculture sector), and cultural characteristics (Google mobility measures). Additionally, as dummy variables, we included the world regions. Both reduced datasets were generated to the best extent, compromising the number of variables and number of countries to have a good mix of high- and low-income countries. Finally, all the variables were standardized before use in models by subtracting the mean and dividing by the standard deviation.

As the dependent variable we used data on the number of COVID-19 deaths from WHO’s COVID-19 Dashboard as the variable indicating the severity of the COVID-19 epidemic outcome in an individual country. All gathered data was prepared in pre-processing step (e.g., logarithmic transformation) and analyzed in order to prepare for estimation of regression models. In the first two models, the least squares method was used. In the third one, the Huber-White-Hinkley estimator was used. We used software package EViews 10+ (Enterprise Edition, 64-bit, IHS Global Inc., Irvine, CA, USA, 2018) for the model estimation.

A limitation to the study has to be noted here. Although we have taken unified data sources across countries, different inconsistencies in the methodology of collecting data can be found, e.g., number of dead due to COVID-19 (as a main source of implication) is not uniquely defined across countries. The full extent of COVID-19 outcome will be possible to be evaluated when all statistical data in full range and reliability will be available.

## 3. Results

**Figure 1:**
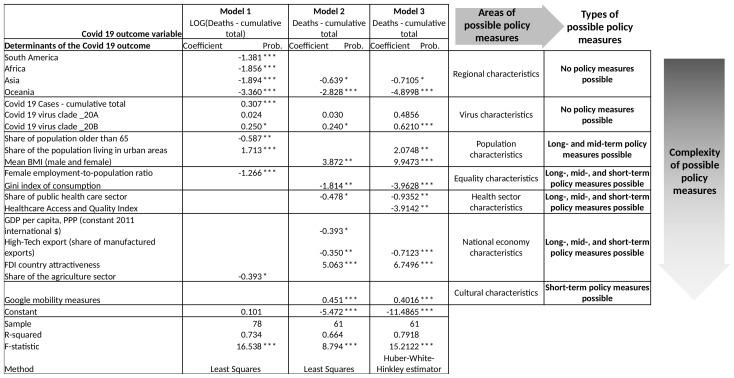
‘*’ = *p*-value lower than 0.10, ‘**’ = *p*-value lower than 0.05, ‘***’ = *p*-value lower than 0.01. Economic policy framework for determining pandemic outbreak measures. Source: own calculations and figure presentation.

Based on empirical evidence, we considered estimations on multiple regression models to draw an integral framework for identification of areas, where the determinants of severity of COVID-19 outcome came from. Although the results depend on the limited selection of countries and variables, both logarithm–linear and linear–linear models suggested reasonably-connotated connections. Multiple models were estimated instead of one, and composite indicators were used. Despite this fact, signs on regression coefficients stayed consistent for explanatory variables that appear in more than one model. The integral framework comprises three models and unites the findings altogether. This is presented in Figure 1. We chose the presented three models over other experimental models as they at best met the criteria of high explanatory power, expressed by high levels of coefficient of determination (R-squared). However, the ability to further improve the study’s econometric quality was impacted by our research aim of including the biggest possible number of countries and the widest possible selection of the explanatory variables. Nevertheless, the final models exhibit high values of R-squared, especially due to the fact that we are dealing with cross-section and highly heterogeneous data.

In this research, there was a challenge of heteroscedasticity. In the modelling phase, we controlled for the heteroscedasticity by different approaches reflected in the three models. In the first, we chose to use the logarithmic value of the dependent variable. In the third, we took another approach, namely, a heteroscedasticity robust estimator: Huber–White–Hinkley estimator. For a benchmark, we did not apply any adjustments in the second model due to the heteroscedasticity.

When interpreting the results, another fact should be taken into account, namely the possible presence of multicollinearity. In the initial modelling step, multicollinearity has impacted the selection of explanatory variables severely. After the selection, we empirically found that a possible threat of multicollinearity was still indicated in the model. Namely, some of the regression coefficients exposed the signs (connotations, i.e., −/+) opposite as expected, which we interpreted exclusively as a consequence of multicollinearity. Still, we followed a common econometric rule that a multicollinearity is not a reason for omitting the model.

There were dummy variables included in the models for regions that statistically significantly deviated from the global average. We believe this is due to the huge differences in the initial position at the beginning of the epidemic in individual countries. However, when considering the robust estimator, the only significant results remain the dummies for Asia and Oceania, where very restrictive measures against the spread of the virus were applied.

The results on the estimated econometric models reveal some interesting findings. Among contributing factors to a more severe epidemic outcome, higher population mobility, a higher level of the population living in urban areas, a weaker physical condition of the population, and the openness of the economy all featured. On the other hand, a positive impact came from a higher share of the primary economic sector in the ecosystem structure (agriculture) and high economic development measured as High-Tech export. Additionally, the importance of public healthcare was revealed, as better healthcare access and quality notably contributed to a more favourable epidemic outcome.

The results suggest there are multiple areas which determined the severity of the COVID-19 outcome in individual countries:

- regional characteristics;
- virus characteristics;
- population characteristics;
- equality characteristics;
- healthcare sector characteristics;
- national economy characteristics;
- cultural characteristics.

When examining the areas closely, the overall analysis of all three models suggests that there are three groups of factors which influence the outcome of the pandemic in individual countries. In regards to the economic policy, these groups differ and can be listed as follows:

- areas where factors cannot be influenced by economic policy measures;
- areas where factors can be influenced by long- and mid-term policy measures;
- areas where factors can also be influenced with short-term policy measures and prompt results are possible.

The analysis of the framework reveals that economic policy measures cannot influence the regional characteristics, e.g., where the individual country is placed, as well as the virus characteristics, e.g., virus clade present in the particular country. The other two groups of factors are relevant for the economic policy, as they might be influenced by long, mid-, and short-term policy measures. The area of population characteristics could be addressed with mid- and long-term measures, and could be directed to the population structure, ranging from living conditions such as urbanization up to ageing structure or physical characteristics of the population. The group of measures, likely to be less complex than those previous, would be long-, mid-, and short-term policy measures and would aim to favourably enhance the equality characteristics of the society. The understanding of equality, in this sense, is broad and includes the gender impact, the labour market conditions, and the distribution of wealth, also on the regional level.

The next area of possible economic policy measures would be undertaken aiming at changes of the healthcare sector characteristics. These can be impacted with combination of long-, mid-, and short-term measures, therefore it also includes structural characteristics of the sector, including the capacity, quality, and accessibility of the services. The area of characteristics of the healthcare sector includes the structure according to the public and private share of the healthcare sector. The results are in favour of a larger share of the public healthcare sector.

Our results also indicate that the characteristics of national economy had an impact on the severity of the pandemic outcome. By economic characteristics, not only the level of economic development measured by e.g., GDP per capita is meant, but the structure of the economy, namely the sectoral structure, is encountered. The level of innovation and structural changes will be at the forefront of this area of economic policy measures.

Furthermore, short-term policy measures could influence the area of cultural characteristics, among which the mobility of the population is limited. The complexity of measure will gradually increase. The less complex measures will be applied at the area of population characteristics, while the most complex measures are expected to be applied at the area of national economy characteristics and the cultural characteristics.

Based on empirical findings, we propose a mix of possible economic policy measures directly or indirectly linked to the healthcare sector. This includes promoting public healthcare, ensuring crisis capacities, and access to quality healthcare. On the other hand, state and obligatory health insurance premiums should also account for individuals’ decisions, resulting in higher healthcare costs, e.g., non-vaccination once a vaccine is available. Alternatively, participation in healthcare costs for non-vaccinated could be applied and used to finance scaling-up the capacities. To encourage a resilient economy for the future, economic policy must implement policy measures, based on empirical findings on characteristics of economic structures and multiplicative effects as well as actual lessons learned from the economic consequences of COVID. Furthermore, it has been argued that standard fiscal stimulus might be less effective than normally expected due to muted Keynesian multiplier feedback (Guerrieri et al. [10]). Additionally, as indicated in the literature (Bekö et al. [15]), the impact of the healthcare sector seems to remain stable throughout the business cycle, which suggests the predictability of economic measures.

In the end, economic recovery is costly. Instead of burdening future generations due to higher public debt, financing sources should be at the cost of individuals who behave opportunistically in the epidemic crisis. State sovereignty includes fiscal measures; therefore, finding these additional sources in a form of a COVID-19 tax could be justified.

## 4. Discussion

As with any study, the limitations have to be considered for proper interpretation of the results. In this study, limitations arise from two perspectives: the data and the methods. Although we have taken unified data sources across countries, we found inconsistencies in their data collection approaches, e.g., number of deaths due to COVID-19 is not uniquely defined across countries. Further, the data availability was limited in the sense that for an individual variable for some countries there were missing values. Consequently, it has led to the trade-off between a larger number of variables or a larger number of included countries. The results are thus impacted by the choice we made in this perspective and might differ from models, where we would either include fewer explanatory variables but even more countries or contrary, more explanatory variables, and fewer countries. Further, regarding the study design, standard testing of policy impact (e.g., treatment effect models, but also Granger causality test) was according to the nature, quality, and availability of data not possible to apply. Additionally, because the pandemic and the applied measures have not yet come to an end, other econometric approaches as what we went for did not seem reasonable in our case. Again, we tried to make the study as broad as possible (in the number of countries included and in the range of variables included), which also impacted the possibilities of applied econometric approaches.

The obtained scientific implications thus are based on a starting period of the COVID-19 pandemic. Later, it will be possible to evaluate the full extent of the dependencies analysed here in relation to COVID-19, once all statistical data in full range and reliability is be available.

The study gives several scientific implications. We found that multiple factors, which determined the severity of the COVID-19 outcome in individual countries, arise from regional, virus, population, equality, healthcare sector, national economy, and cultural characteristics.

Along with the scientific implications presented in detail in the results section, another important finding was revealed by this study, namely the relevance of high-frequency data. In our study, we used Google mobility data as one explanatory variable, but many more could be relevant in the future. High-frequency data, in general, emerged as a result of the use of modern information technologies. However, two aspects of their applicability in science have to be given attention: first, appropriate methodological approaches capable of dealing with such data, and secondly, the availability of the data to the scientific community.

Next, we turn to the economic policy framework, which is serving as an identification matrix for policy implications. We identified several areas that could be relevant for the severity of the epidemic outcome. This section discusses several ideas that suggest economic policy measures to impact the severity of the epidemic outcome favourably.

Our results suggest that national economy characteristics matter; thus, we discuss the policy measures which would address them. The GDP per capita and high-tech export could be influenced. Financial data show that the healthcare sectors’ stocks outperformed most others. The research and development in the healthcare sector industry promotes a high level of innovation which not only contributes to the affordable healthcare, but promotes economic development with high value-added and creates jobs for highly skilled professionals. Encouraging investments in innovative industries (healthcare, pharmaceutical, biotech, and associated industries) could thus be a good way to influence the variables which are found in the group “national economy characteristics” in our framework.

Next, we argue that post COVID-19 investments should encourage R&D in artificial intelligence (AI). Innovation and transformation accelerate economic growth and promote resilient economic systems. AI can already be applied as the first stage in diagnosing less severe cases, thereby releasing capacities (AbuShaban [2]). Investing in AI in the healthcare sector will have huge spill over effects, as this means investment into AI professionals, companies developing AI solutions, and implementation of these solutions in other sectors, making the economy future-ready. Promoting R&D in AI and AI usage in healthcare could have a favourable impact on the variables in the groups “national economy characteristics” as well as “health sector characteristics”. Additionally, AI can be seen as a convenient tool for stipulation of a healthy lifestyle (in smart watches, sensors, and wearables), which importantly lowers COVID-19 severity (we noticed a significant connection between physical condition measured by BMI and cumulative total).

We further discuss the measures aiming to change the characteristics of the healthcare sector, especially due to the high relevancy of the regression variable “Healthcare Access and Quality Index” (−3.9142 **), as indicated in the third model of the variable. Additionally, the framework from this study indicates that the private–public healthcare matters. As the healthcare sector must be part of the critical infrastructure, the government and the private sector should establish a relationship between each other to encourage the necessary cooperation (see also AbuShaban [2]). Networks of regional providers are more critical to community recovery than centres.

Public healthcare providers are more suitable to provide sufficient backup capacities in areas which are not profitable. If public healthcare providers operate in profitable healthcare services, profits can be used for covering losses from operating in non-profitable services. For example, reserving and maintaining capacities for national medical emergencies is costly and does not gain profits.

Transformation of healthcare systems with more flexibility can contribute to provide access to quality healthcare. Both flexibility in physical capacities (AbuShaban [2]) and medical staff flexibility (Ferreira et al. [34], Casha and Casha [35]) should thus be addressed.

The healthcare sector of many countries is suffering from medical stuff shortages resulting from emigration of medical professionals (Ferreira et al. [34], Casha and Casha [35]). We argue that there is the need to design policy measures that mitigate the intention of healthcare professionals to emigrate. Temporary deficits on the labour markets can be solved by encouraging short-term medical staff mobility. Long-term shortages should be addressed by economic policy measures. However, one must notice that mobilities in general are not appreciated, as the “Google mobility measures” exposes positive regression coefficients.

Additionally, we suggest reconsidering “state aid” in industries that negatively affect health and environment in any economic policy action. Therefore, capital injection measures should be considered in industries according to economic, environmental, and health criteria. This would have long-term effects on health and environment and would make economies more resilient to future disruptions while also contributing to equity.

## 5. Conclusions

In this study, we support the thesis that innovative measures of economic policy should be applied in the after-COVID-19 period. These measures should differ from traditional ones, be applied in advance of an epidemic, and thus to support economic and social ecosystems to become more resistant to current and future epidemic crises. Many of the proposed measures directly or indirectly concern the healthcare systems and healthcare sector, aiming to impact its characteristics, such as public-vs-private healthcare or the access and quality of the healthcare provided. Economic policy measures could thus promote new technologies in healthcare, sectoral staff flexibility, or reinforce decentralised (regional) public health services providers. A central element of this study, the innovative identification matrix, which combines unbiased econometric results with remediation, could be populated as a unique policy framework, either for latest pandemic or any similar outbreaks in future. However, such a policy framework is not only to be used for identifying pandemic outcomes, but also when the final data on the pandemic outbreak outcomes become available, to make accurate and reliable predictions of the effect on individual economic, health, and social life factors. In the end, its application in policy design could contribute to modern societies’ efforts on equality, human rights, and social cohesion.

We suggest further research on this topic. With the passage of time, the data on the longer time frame of the COVID-19 pandemic period will be available. This would enable a panel-based econometric approach instead of a cross-sectional one. In doing so, both the range of included characteristics of the countries and the genetic changes of the virus could improve the model and reveal new dependencies in the examined countries’ characteristics to the severity of the pandemic. Additionally, as we discussed the scientific potential of high-frequency data, future research could include them in investigations. The latter would enable the detailed study of interaction between high-frequency data variables and the genetic profile of the virus.

## Data Availability

Publicly available datasets from multiple sources were analysed in this study. The data can be found here: [https://data.worldbank.org/, https://www.imf.org/en/Publications/WEO/weo-database/2020/October, https://tradingeconomics.com/indicators, http://www.fdiattractiveness.com/ranking-2020/, https://yearbook.enerdata.net/, https://www.itu.int/en/ITU-D/Statistics/Pages/stat/default.aspx, https://kof.ethz.ch/en/forecasts-and-indicators/indicators/kof-globalisation-index.html, https://www.google.com/covid19/mobility/, https://apps.who.int/gho/data/node.main, https://nextstrain.org/ncov/global, https://ourworldindata.org/charts], all accessed on 10 December 2020.
